# UPLC-QTOF-MS-Based Metabolomics and Antioxidant Capacity of *Codonopsis lanceolata* from Different Geographical Origins

**DOI:** 10.3390/foods12020267

**Published:** 2023-01-06

**Authors:** Miso Nam, Sae rom Jo, Young-Chan Kim, Min-Sun Kim

**Affiliations:** 1 Food Analysis Research Center, Korea Food Research Institute, Wanju 55365, Republic of Korea; 2 Enterprise Solution Research Center, Korea Food Research Institute, Wanju 55365, Republic of Korea

**Keywords:** antioxidant capacity, bioactive compound, *Codonopsis lanceolata*, geographical origin, metabolomics

## Abstract

*Codonopsis lanceolata* (*C. lanceolata*) has been commonly utilized as a therapeutic plant in traditional medicine. In this study, we examined variations in metabolites in *C. lanceolata* roots grown in different regions using ultra-high performance liquid chromatography quadrupole time-of-flight mass spectrometry (UPLC-QTOF-MS). Multivariate analysis showed that the metabolite profiles of plants grown in Hoengseong and Jeongseon were more similar to each other than to that of *C. lanceolata* grown in Jeju. Most primary metabolites were present at higher levels in *C. lanceolata* grown in Jeju. In contrast, *C. lanceolata* grown in Hoengseong and Jeongseon had high levels of secondary metabolites such as phenylpropanoids and triterpenoid saponins, respectively. In addition, the bioactive compound content and antioxidant capacity of in *C. lanceolata* grown in Hoengseong and Jeongseon were observed to be higher than those of *C. lanceolata* grown in Jeju. This study suggests that metabolomics is an effective approach to investigate the difference of metabolite profiling in *C. lanceolata* from different geographical origins, and is useful for evaluating its pharmacological potential.

## 1. Introduction

*Codonopsis lanceolata* (*C. lanceolata*) is a perennial twining vine that is classified in the Campanulaceae family and *codonopsis* genus. It is widely distributed or cultivated in East Asia, such as South Korea, China, and Japan [1]. *C. lanceolata* is attracting interest due to its unique flavor and taste, and its roots and leaves are edible raw or cooked. *C. lanceolata* is composed of various bioactive components, including alkaloids, phenylpropanoids, polyphenols, saponins, and steroids [2]. *C. lanceolata* extract has been reported to have potential therapeutic effects against obesity, oxidative stress, inflammation, diabetes, and cancer and is associated with cognitive improvement [3,4,5,6]. Additionally, in oriental medicine, *C. lanceolata* has been used as a medicinal herb to treat various lung inflammation-related symptoms such as cough, sputum, and bronchitis by increasing lung energy [7]. *C. lanceolata* has been used as a substitute for ginseng because it has a comparable effect, and its demand is gradually increasing because it is less expensive than ginseng [7,8,9]. Although *C. lanceolata* consumption is increasing due to its various pharmacological effects on human health, knowledge regarding its major components and biological function is insufficient compared to that of other similar plants.

Because plant metabolite compositions are influenced by environmental conditions such as temperature, soil conditions, and precipitation [10], metabolite composition is a good criterion for determining food quality and geographical origin. Plant metabolomics can be used to simultaneously determine primary and secondary plant metabolites using various analytical techniques such as mass spectrometry (MS) combined with gas chromatography or liquid chromatography (LC), and nuclear magnetic resonance [10,11]. Thus, metabolomics has the advantage of efficiently revealing changes in metabolites within a short time. Ultra-high performance liquid chromatography (UPLC)-MS techniques are rapid and have high sensitivity and chromatographic resolution, facilitating the full analysis of complex samples with a broad range of metabolites [12]. Recently, metabolomics studies of *C. lanceolata* have been conducted, and a UPLC-quadrupole time-of-flight-MS (UPLC-QTOF-MS)-based metabolomics study evaluating *C. lanceolata* from various regions of China was reported [13]. Although a previous metabolomic study demonstrated the discrimination of *C. lanceolata* grown in regions of South Korea, the selected areas are not major producing regions of *C. lanceolata* in South Korea [14].

In this study, we conducted an untargeted metabolomics using UPLC-QTOF-MS to determine the metabolite profile of *C. lanceolata* grown in three major production regions in South Korea: Jeju (JJ), Hoengseong (HS), and Jeongseon (JS). Additionally, we analyzed and compared the bioactive compound content and antioxidant capacity of *C. lanceolata* grown in each region to determine its pharmacological characteristics by region. Our study provided information on the metabolite characteristics influenced by the cultivation environment of *C. lanceolata*, and may contribute to understanding whether a positive correlation exists in the relationship between the antioxidant capacity and metabolite profile.

## 2. Materials and Methods

### 2.1. Sample Materials and Chemicals

*C. lanceolata* samples were collected from three regions which are Jeju, Hoengseong, and Jeongseon in South Korea. Samples were obtained from local markets, and each sample was grown on a different farm. *C. lanceolata* samples were harvested between August and October 2021. Table 1 shows the basic and geographic information of *C. lanceolata* samples, and Figure S1 shows geographical locations and representative samples of *C. lanceolata*. After washing several times in running water, the skin was removed. It was freeze-dried for 72 h, at −50 °C condenser temperature and a pressure of 10 mTorr using a freeze dryer (FDCF-12003, Operon, Korea). After lyophilization, the samples were finely powdered and stored at −80 °C.

LC-MS grade acetonitrile, water and formic acid and HPLC grade ethanol were purchased from Fisher Scientific (Pittsburgh, PA, USA). 2,2-diphenyl-1-picrylhydrazyl (DPPH) were purchased from Cayman Chemical (Ann Arbor, MI, USA)

### 2.2. Preparation of Samples for UPLC-QTOF-MS Analysis

For metabolites extraction, 1 mL of water/ethanol (30:70, *v*/*v*) was added to 50 mg of dried *C. lanceolata* as extraction solvent. The sample was mixed for 2 min and ultrasonicated for 20 min at room temperature. The extracted solution was then centrifuged at 12,000× *g* rpm for 20 min, and the supernatant solution was filtered through a 0.2 μm PTFE syringe filter (Millipore, Billerica, MA, USA) and transferred to LC-MS vial.

### 2.3. UPLC-QTOF-MS Analysis and Data Processing

An UPLC-QTOF-MS analysis were performed on an Exion LCTM AD system (AB SCIEX, Toronto, Canada) connected to a X500R QTOF system. The chromatography Chromatographic separation was implemented using an Acquity UPLC HSS T3 column (2.1 mm × 100 mm, 1.8 μm; Waters, Milford, MA, USA) at a temperature of 40 °C; The mobile phases consisted of (A) water with 0.1% formic acid and (B) acetonitrile 0.1% formic acid. The linear gradient was started as follows: 1–5% B (0–1 min), 5–25% B (1–3 min), 25–35% B (3–4.8 min), isocratic 35% B (1 min), 35–45% B (5.8–6.8 min), isocratic 45% B (1 min), 45–60% B (7.8–8.8 min), 60–100% B (8.8–9.3 min), 100–1% B (9.3–10 min), and 1% B (10–13 min). A flow rate of 0.3 mL/min, the injection volume was 5 μL and the temperature of the autosampler during analysis was at 4 °C. The mass range was from 100 m/z to 1300 m/z and data acquired both in positive and negative ionization modes. The following experimental parameters were used for the operation: ion spray voltage, 5500 V (ESI+) or −4500 V (ESI−); source temperature, 500 °C; declustering potential, 90 V; nebulizer gas pressure, 50 psi; and curtain gas pressure, 30 psi. IDA was performed to acquire MS/MS spectrum with the following parameters: collision energy, 40 V; collision energy spread, 15 V. All samples of equal volume were pooled to generate a quality control (QC) sample. QC samples were analyzed before running sample acquisition and after every nine samples. MarkerView (AB Sciex) was employed to find peaks, perform the alignment and generate peak list of m/z and retention times (min).

### 2.4. Preparation of the Ethanolic Extract of C. lanceolata

20 g of dried *C. lanceolata* was extracted with 400 mL of water/ethanol (30:70, *v*/*v*) at 70 °C for 4 h using a reflux condenser and cooled. The undissolved residues were filtered through filter paper (No. 2, ADVANTEX, Tokyo, Japan). After filtration, the sample was concentrated by a vacuum evaporator. The residue was freeze-dried and dried extracts were stored at −80 °C.

### 2.5. Determination of Bioactive Compounds

The total phenolic content (TPC) was determined according to the modified Folin–Ciocalteu method [15]. The extracts diluted with water/ethanol (30:70, *v*/*v*), were reacted with 0.2 N Folin-Ciocalteu reagent. After 6 min, the 10% sodium carbonate solution was added. After 1 h, the absorbance was measured at 765 nm. The calibration curve of gallic acid was used to calculate the TPC in the extract of *C. lanceolata* and the calibration curve equation was y = 0.0102x − 0.2243 (r^2^ = 0.9994). The results were expressed as mg of gallic acid equivalents (GAE)/100 g of dry extracts. Synergy H1 microplate reader (Bio Tek, Winooski, VT, USA) was used to measure the absorbance.

The total flavonoid content (TFC) was measured based on the aluminum chloride colorimetric method [16]. The extracts in water/ethanol (30:70, *v*/*v*) were successively reacted with 5% sodium nitrite and distilled water for 5 min, with 10% aluminum chloride for 6 min, and with 1 M sodium hydroxide for 11 min. The absorbance was measured at 420 nm. The calibration curve of quercetin was used to calculate TFC in the extract of *C. lanceolata* and the calibration curve equation was y = 0.0013x + 0.0142 (r^2^ = 0.9955). The results were shown as mg of quercetin equivalents (QE)/100 g of dry extracts.

### 2.6. Biological Assays

#### 2.6.1. Cell Culture

HepG2 cells were maintained in Dulbecco’s modified Eagle’s medium (DMEM) supplemented with 100 U/mL penicillin, 100 μg/mL streptomycin, and 10% fetal bovine serum (FBS) at 37 °C in a humidified atmosphere of 5% CO_2_. The cells were obtained from the Korea Cell Line bank (KCLB, Seoul, Korea).

#### 2.6.2. Determination of Intracellular ROS Scavenging Activity

Intracellular ROS levels were measured by the dichlorodihydrofluorescein diacetate (DCF-DA) assay [17]. To investigate intracellular ROS levels, hepG2 cells were seeded in a black 96-well plate at a density of 2 × 10^4^ cells/well. After 24 h, the medium was replaced with 10 μM DCF-DA (dissolved in DMSO) for 10 min at 37 °C in the dark. After washing out the excess probe with PBS, the cells were treated with the DW for control, or extracts (500 μg/mL) of *C. lanceolata* in the presence of 500 μM H2O2. After 40 min, fluorescence was detected by microplate reader using the λ (ex./em.) = 480/530 nm.

### 2.7. Determination of Antioxidant Capacity

#### 2.7.1. DPPH Radical Scavenging Assay

The DPPH free radical scavenging activity of *C. lanceolata* extracts was evaluated by a method modified from a previous study [15]. 100 μL of different concentrations of sample solutions was added to 96-well plates, with the addition of DPPH solution (0.2 mmol/L, 100 μL). The mixture was shaken and left the plate at room temperature in the dark. After 5 min, the absorbance was measured at 517 nm.

#### 2.7.2. Ferric Reducing Antioxidant Power (FRAP) Assay

The FRAP assay was evaluated by using the MAK369-1KT kit (Sigma, St Louis, MO, USA). The procedure was performed according to the manufacturer’s instructions. After the samples were added into each 96-well plates, the assay buffer and the working solution were added and incubated at 37 °C. After 1 h the absorbance was measured at 594 nm.

#### 2.7.3. Antioxidant Assay

Total antioxidant capacity of the extracts was measured using the antioxidant assay kit (CS0790, sigma), according to the manufacturer’s protocol. Trolox was used as a standard. After the samples were added into each of the 96-well plates, myoglobin working solution and ABTS (2,2′-azino-bis 3-ethylbenzthiazoline-6-sulfonic acid) substrate working solution were added. All samples were then incubated at room temperature for 5 min. The reaction was stopped by adding stop solution (100 μL) to each well. The endpoint absorbance was measured at 405 nm by a microplate reader.

### 2.8. Statistical Analysis

Multivariate statistical analyses were performed with a unit variance scale using SIMCA-P+ software, version 17.0 (Umetrics, Umeå, Sweden). The statistical significance of the data was analyzed by Kruskal–Wallis test, followed by Dunn’s post hoc test using GraphPad Prism (San Diego, CA, USA). A hierarchical cluster analysis (HCA) and heat map were generated using MetaboAnalyst Ver. 5.0.

## 3. Results and Discussion

### 3.1. Metabolite Profiling of C. lanceolata Extracts Using UPLC-QTOF-MS

*C. lanceolata* samples collected from three different major *C. lanceolata*-producing areas in South Korea, JJ, JS, and HS, were subjected to extraction and analysis. The factors contributing to the metabolite composition of *C. lanceolata* from different origins can be complex and may be influenced by environmental factors, such as climate, soil type, and fertilization. The JS and HS areas are in the Gangwon Province, South Korea, located in the central part of the Korean peninsula, are geographically close, and have more similar environmental factors, such as climate and soil type, to each other than to those of JJ. JJ is an island located in the southernmost part of South Korea. JS and HS have a combined mountain and continental climate and have cooler weather conditions; the average annual temperatures in these areas are 10.7 °C and 11.3 °C, respectively. Compared to the other two regions, JJ has a milder environment, with an average annual temperature of 16.5 °C, and it does not have a large annual temperature range. JS and HS are representative limestone regions, and JJ is a volcanic region. These distinct climatic and soil type factors may significantly influence the metabolite profile of *C. lanceolata*. In addition, the *C. lanceolata* produced in the three regions are of different ages. *C. lanceolata* cultivated in JJ, a temperate region, is harvested within 2–3 years. However, 5–6 years are required to cultivate *C. lanceolata* in JS, where it is cultivated at a high altitude, similar to the environment where wild *C. lanceolata* grows, at an elevation of 300 m or higher. This difference in maturity may be a factor that affects the differentiation of metabolite profiles in *C. lanceolata*.

To investigate differences in the metabolite composition of *C. lanceolata* cultivated in different areas, untargeted metabolic profiling of *C. lanceolata* extracts was conducted. Root extracts were analyzed by UPLC-QTOF-MS. Representative total ion chromatograms of the extracts from positive and negative UPLC-QTOF-MS ionization modes are shown in Figure S2.

To investigate the discriminatory characteristics and visualize metabolic differences among extracts of *C. lanceolata* grown in different areas, unsupervised principal component analysis (PCA) was performed with the mass spectra of *C. lanceolata,* and the results are presented in Figure S3. The PCA model was established using the positive and negative modes (positive, R^2^ = 0.611, Q^2^ = 0.335; negative, R^2^ = 0.578, Q^2^ = 0.328). The use of QC samples to assess the precision of detection is a common practice in untargeted metabolic profiling. QC samples were closely clustered in the PCA score plots obtained from positive and negative mode spectra, indicating good stability of the UPLC-QTOF-MS analysis throughout the experiment. The PCA score plots showed that some HS and JS samples partially overlapped with JJ samples, but the three groups of samples could be distinguished under unsupervised conditions.

In the present study, 92 metabolites were putatively identified in *C. lanceolata* samples from different geographical origins based on their molecular formula. The major classes of identified metabolites were organic acids, amino acids and derivatives, sugars, glycosides, triterpenoids, polyacetylenes, phenylpropanoids, flavonoids, and others. These metabolites are detailed in Table S1. Metabolites were identified as amino acids, organic acids, sugars, and phenylpropanoids by comparing the mass accuracy of precursor ions and their similarity of MS/MS spectra patterns to those in freely accessible metabolite databases, including the Human Metabolome Database and METLIN database. Other metabolite classes, such as glycosides, triterpenoids, polyacetylenes, phenylpropanoids, and flavonoids, were tentatively identified by comparing the exact mass of precursors and fragment ions with previously published data [4,14,18,19,20,21,22,23,24,25].

**Figure 1:**
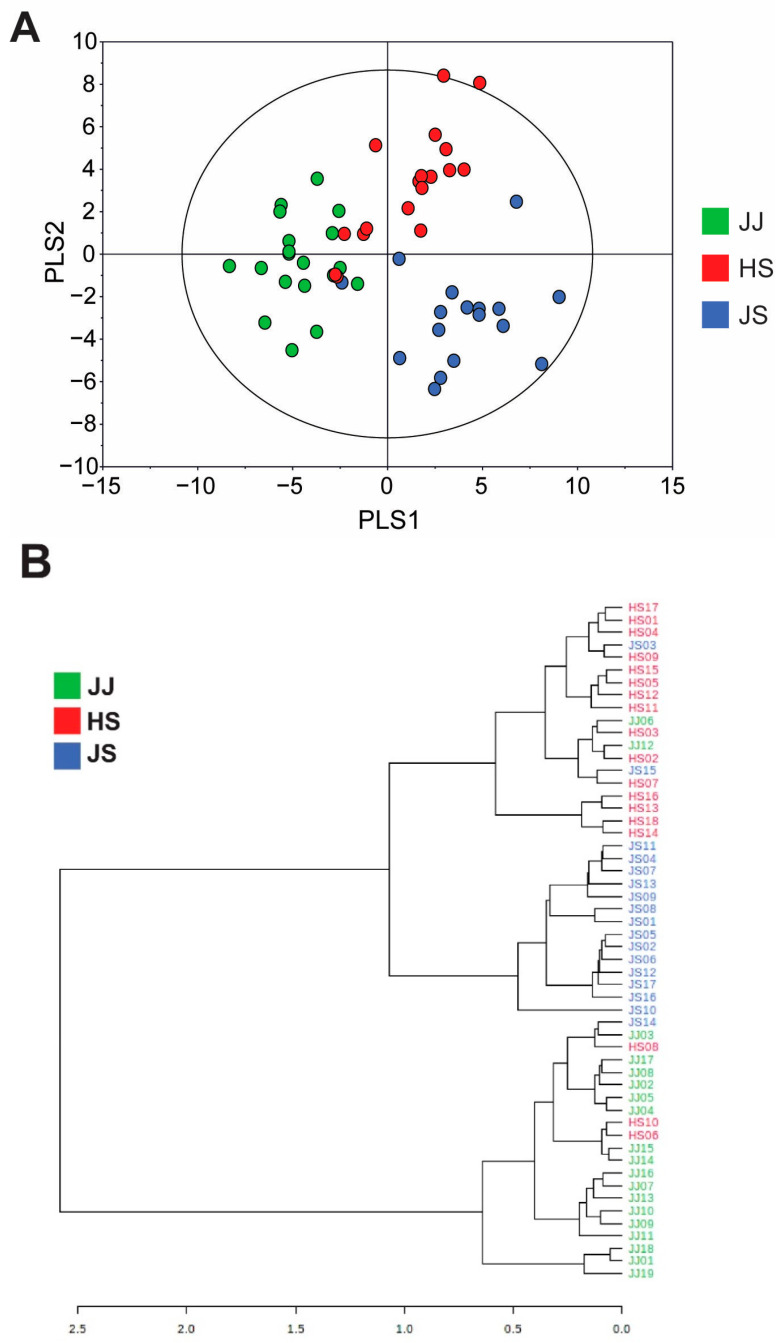
PLS-DA score plot (**A**) and HCA (**B**) derived from identified metabolites from *C. lanceolata* samples obtained from three different geographical origins.

To remove the many irrelevant variables and further our understanding of the identified metabolite patterns in *C. lanceolata* roots, partial least squares-discriminant analysis (PLS-DA), which can maximize the separation between observation groups and improve classification and prediction capabilities, was performed with the identified metabolites. The PLS-DA score plot shows separation patterns among the three groups (Figure 1A, R^2^X = 0.344, R^2^Y = 0.634, Q^2^ = 0.554). The JJ group was mainly separated from the HS and JS groups along the PLS1 axis, and the HS and JS groups were separated by the PLS2 axis. Variation within each group can be attributed to the fact that each sample was grown on a different farm, as each farm had different fertilizers, storage conditions after harvest, age of *C. lanceolata*, and harvest time. HCA, an unsupervised chemometric method, was also performed with the metabolites identified in *C. lanceolata* samples to determine the degree of similarity between the groups. A small distance indicates a high degree of association. As shown in Figure 1B, although some samples were located between groups, the samples were separated into two large groups: a group including samples from HS and JS, and another group with samples only from JJ. The multivariate analysis showed differences in the metabolite profiles of *C. lanceolata* grown in the three regions, although there might be variation within each group due to other factors except geographical origin. The metabolite profiles of *C. lanceolata* grown in HS and JS were more similar to each other than to that of *C. lanceolata* grown in JJ.

### 3.2. Differences in C. lanceolata Metabolites According to Geographical Origin

**Figure 2:**
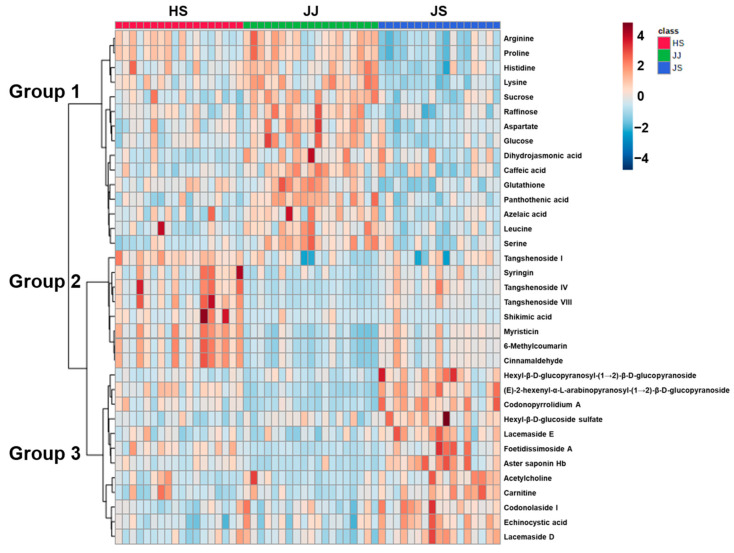
A heat map of metabolites with significant *p*-values (*p* < 0.001) by Kruskal–Wallis analysis.

Metabolites that significantly contributed to the differentiation among *C. lanceolata* samples from different origins were determined using univariate statistical analysis. A total of 35 metabolites with significant *p*-values using the Kruskal–Wallis test (*p* < 0.001) were visualized as a heat map, and these metabolites were divided into three groups that showed different concentration patterns (Figure 2). Group 1 metabolites were most abundant in samples from the JJ region, while group 2 and group 3 compound levels were highest in the HS and JS region samples, respectively. In addition, group 2 and group 3 metabolites could be merged into one of the two main clusters, which again showed that a small metabolite profile gap existed between JS and HS samples and that JJ samples had the most distinct metabolite profile.

**Figure 3:**
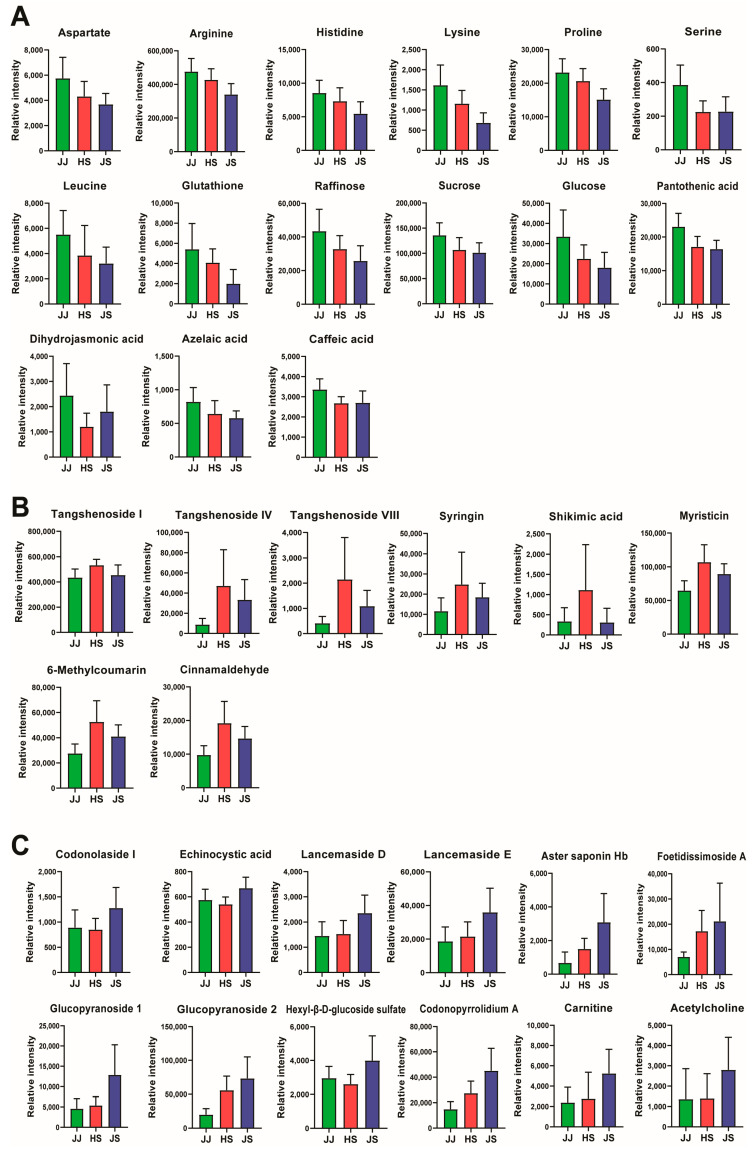
Bar graphs of the normalized peak intensity of significantly different metabolites belonging to group 1 (**A**), group 2 (**B**), and group 3 (**C**) of Figure 2 in *C. lanceolata* samples obtained from three different geographical origins. The results are expressed as mean ± SD. Glucopyranoside 1 is hexyl-β-D-glucopyranosyl-(1→2)-β-D-glucopyranoside and glucopyranoside 2 is (E)-2-hexenyl-α-L-arabinopyranosyl-(1→2)-β-D-glucopyranoside.

The metabolites in group 1 were mainly primary metabolites (Figure 3A), such as amino acids and their derivatives (arginine, proline, histidine, lysine, aspartate, leucine, serine, and glutathione), sugars (sucrose, raffinose, and glucose), organic acids (dihydrojasmonic acid and azelaic acid), and vitamins (pantothenic acid). Plant primary metabolites help to carry out the basic functions of living cells, thus substantially contributing to growth and agricultural yields [26]. The high content of primary metabolites in *C. lanceolata* from JJ allows it to grow in 2–3 years to a size similar to that of *C. lanceolata* grown in JS for 5–6 years.

The concentrations of free amino acids in plant tissues are related to the nitrogen supply, and an abundant nitrogen supply increases the total free amino acid concentration in many plant species [27]. A previous study reported that amino acid synthesis and concentrations in soybean leaves grown in the JJ area were increased compared with those grown in Yeongwol, because the soils of JJ have greater levels of organic matter and nitrogen than those in the Yeongwol area near HS and JS, which belong to Gangwon Province [28]. The abundant nitrogen supply in the soil of the JJ area would have caused an increase in amino acid levels in *C. lanceolata* samples from JJ.

In the present study, elevated levels of sugars such as glucose, sucrose, and raffinose were found in *C. lanceolata* roots grown in JJ compared with those grown in HS and JS. Factors such as temperature and year of cultivation can affect sugar levels in plants. Root respiration rates decrease at low temperatures, which is negatively correlated with reducing sugar concentration [29]. In a previous study, sugars such as fructose, glucose, and sucrose in ginseng roots were abundant in samples collected at 3 and 4 years compared with those collected at 6 years [30]. Although the temperature in JJ is higher than that in HS or JS, the high sugar concentration in *C. lanceolata* from JJ may have been influenced by the short cultivation year.

Most group 2 metabolites exhibited the highest content in the HS group and were secondary plant metabolites, including phenylpropanoids (Figure 3B). As one of the most critical metabolic pathways in plants, the phenylpropanoid pathway contributes to plant development and environmental interactions [31].

Syringin, a bioactive compound, has pharmacological significance because of its antioxidant and anti-inflammatory effects [32]. Syringin were detected at *m/z* 390 from [M + NH4]^+^ and fragment ions at *m/z* 161 [18]. Tangshenoside, which is considered a syringin molecule bound to meglutol glucoside, is the major constituent of the genus *codonopsis*. Tangshenosides I showed [M − H]^−^ ion at *m/z* 677 and generated fragment ions at *m/z* 497 [M − H − C_6_H_12_O_6_]^−^, *m/z* 453 [M − H − C_6_H_12_O_6_ − CO_2_]^−^, due to the loss of one glycosyl moiety and a successive decarboxylation, and *m/z* 261 [C_11_H_17_O_7_]^−^ [20]. Tangshenosides I, IV, and VIII have been found in the roots of *C. lanceolata* [13,20,24]. In vitro and in vivo studies in mice have shown that tangshenoside I contributes to ameliorating skeletal muscle atrophy via activating the PI3K/Akt/mTORC1 pathway and upregulating the SIRT1/PGC-1α pathway [33].

Myristicin, 6-methylcoumarin, and cinnamaldehyde are also types of phenylpropanoids. Myristicin is effective in treating diarrhea, abdominal pain, and anxiety and is known to possess anti-inflammatory activity in the inflammatory response [34]. 6-Methylcoumarin inhibits inflammation by downregulating the MAPK and NF-κB pathways as a potential treatment for inflammatory diseases [35]. Cinnamaldehyde is a potential antidiabetic compound with both hypoglycemic and hypolipidemic effects, that affect glucose concentration and decreases glucolipid levels in streptozotocin-induced diabetic rats [36].

Most group 3 metabolites were triterpenoid saponins (codonolaside I, echinocystic acid, lancemaside D, lancemaside E, aster saponin Hb, and foetidissimoside A) and had the highest abundance in *C. lanceolata* roots grown in JS (Figure 3C). Lancemaside D, aster saponin Hb, and foetidissimoside A showed [M − H]^−^ ion at *m/z* 1087, 925, and 1057, respectively, and a product ion *m/z* 647 were generated by neutral loss of a tetrasaccharide unit (two xyloses, arabinose, and rhamnose) [19]. Triterpenoid saponins are plant secondary metabolites with a structure derived from the precursor oxidosqualene, with one or more sugar residues added [37]. More than 10 triterpenoid saponins have been identified from *C. lanceolata* [4]. The saponins found in *C. lanceolata* are considered mainly responsible for their anti-inflammatory properties [6,25], and their stress regulating and radical scavenging actions [38].

Overall, the JJ group had a high primary metabolite content, and the HS and JS groups had a high secondary metabolite content, although differences in metabolite classes were observed. Environmental stresses such as high salinity, drought, ultraviolet irradiation, nutrient deficiencies, and extreme temperature increases the accumulation of secondary metabolites, which overcomes the stressful condition and allows the plant to adapt to the environment [39,40]. Environmental factors such as the low temperature and low nitrogen supply in the soil compared to that in JJ may have acted as environmental stresses, resulting in secondary metabolites accumulation in *C. lanceolata* grown in HS and JS. These differences in secondary metabolite content may also cause differences in the amount of bioactive compounds (flavonoids and phenolics) that contribute significantly to antioxidant capacity.

### 3.3. Determination of the Bioactive Compound Content in C. lanceolata

To investigate the relationship between the secondary metabolite contents and the bioactive compound, we measured the TPC and TFC of ethanolic extracts of *C. lanceolata* related to its antioxidant capacity (Table 2). Phenolic and flavonoid compounds in plants significantly contribute to their antioxidant capacities [41] and are influenced by many factors, including origin, maturation, and climate conditions [42]. The TPC content was greatest in ethanolic extracts of the JS group (426.98 ± 70.05 mg GAE/100 g dry weight), followed by that in the HS group (409.62 ± 42.04 mg GAE/100 g dry weight) and the JJ group (349.98 ± 71.70 mg GAE/100 g dry weight). The TFC content in ethanolic extracts of *C. lanceolata* was also highest in the JS group (281.00 ± 42.50 mg QE/100 g dry weight), followed by the HS group (256.94 ± 33.30 mg QE/100 g dry weight) and the JJ group (185.49 ± 32.61 mg QE/100 g dry weight). Regarding the metabolomics results, *C. lanceolata* in the JS and HS groups had a high amount of secondary metabolites, and similarly, the TPC and TFC contents were higher in *C. lanceolata* grown in JS and HS than that grown in JJ.

### 3.4. Determination of Antioxidant Capacity

To determine the antioxidant capacity of ethanolic extracts of *C. lanceolata*, DPPH radical scavenging, FRAP assays, and antioxidant assays were performed. Table 3 shows the antioxidant activity of *C. lanceolata* grown in the three different areas. When the radical scavenging activity was determined using the DPPH assay, the highest DPPH radical scavenging effect was found in HS groups (IC_50_ = 13.89 ± 2.03), and the lowest DPPH radical scavenging effect was found in JJ groups (IC_50_ = 20.04 ± 5.72). The FRAP results ranged from 32.06 to 22.53 µmol Ferrous equivalents (FE)/g dry weight, and it is the highest in HS groups and lowest in JJ groups, respectively. The antioxidant assay results ranged from 10.14 to 8.19 µmol Trolox equivalents (TE)/g dry weight. The highest were found in the JS and the lowest values were found in JJ groups. This result may have occurred because of the high content of the bioactive compounds TPC and TFC in the HS and JS groups, and the secondary metabolite levels were also high in these two groups.

### 3.5. Cellular Antioxidant Capacity in H_2_O_2_-Stimulated HepG2 Cells

**Figure 4:**
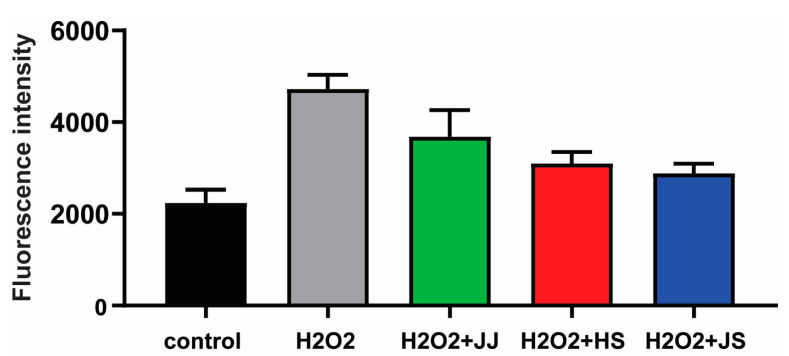
Effects of *C. lanceolata* extracts on the production of ROS in HepG2 cells. The reuslts are expressed as mean ± SD. The experiment was performed in triplicate.

We evaluated the cellular antioxidant activities of *C. lanceolata* with different geographic origins using DCF-DA in H_2_O_2_-treated HepG2 cells. The changes in intracellular ROS fluorescence are shown in Figure 4. When the cells were treated with 500 μM H_2_O_2_, a more than 2.1-fold increase in ROS production compared with that in the control sample was observed. Treatment with *C. lanceolata* from JJ, HS, and JS effectively reduced the H_2_O_2_-mediated production of ROS. This result indicates that *C. lanceolata* grown in all three regions has excellent antioxidant capacity. Among them, *C. lanceolata* from JS removed ROS most effectively, and *C. lanceolata* from JJ had the weakest antioxidant ability. These results support the correlations between the abundance of secondary metabolites and bioactive compounds, and potent antioxidant capacity.

## 4. Conclusions

In the present study, ethanolic extracts from *C. lanceolata* grown in different regions were characterized according to UPLC-QTOF-MS-based metabolomics data and their antioxidant capacity. *C. lanceolata* grown in different regions yielded significant differences in metabolic profiles, bioactive compound content, and antioxidant capacity. We performed metabolite profiling using UPLC-QTOF-MS for the first time in *C. lanceolata* grown in the main production area in South Korea, and a total of 92 metabolites were identified in *C. lanceolata* roots. We found that levels of most primary metabolites were higher in *C. lanceolata* grown in JJ, and that *C. lanceolata* grown in HS and JS was rich in secondary metabolites, including phenylpropanoids and triterpenoid saponins, respectively. In addition, a high bioactive compound content and strong antioxidant capacity were observed in *C. lanceolata* grown in HS and JS. These results indicate that health-beneficial secondary metabolites content in *C. lanceolata* is proportional to the antioxidant capacity of *C. lanceolata*. A limitation of our study is that the age of *C. lanceolata* grown in each region was not consistent. The age of commercially distributed *C. lanceolata* from various regions differs due to its different growth rates depending on the environment, which may have had a critical impact on the metabolite composition. Further studies are needed to discriminate the differences in metabolite profiles caused by environment and maturity. Nevertheless, we determined the metabolite characteristics of *C. lanceolata* grown in each region, thereby providing information about the production environment conducive to *C. lanceolata* quality and pharmacological value.

## Figures and Tables

**Table 1:** Basic and geographic information of *C. lanceolata* samples used in this study.

Area	Coordinates (N,E)	Average Annual Temperature	Age (Years)
Jeju	33°29′58.636″ N 126°31′52.277″ E	16.5 °C	2–3
Jeongseon	37°22′50.718″ N 128°39′39.424″ E	10.7 °C	5–6
Hoengseong	37°29′30.325″ N 127°59′5.748″ E	11.3 °C	3–4

**Table 2:** Bioactive components of the ethanolic extract in *C. lanceolata*.

Bioactive Compounds	*C. lanceolata*			*p*-Value
	JJ	HS	JS	
TPC (mg GAE/100 g dry weight)	349.98 ± 71.70	409.62 ± 42.04 *	426.98 ± 70.05 ^##^	0.0057
TFC (mg QE/100 g dry weight)	185.49 ± 32.61	256.94 ± 33.30 ****	281.00 ± 42.50 ^####^	<0.0001

The results are presented as mean ± SD. The results are presented as mean ± SD. *p*-values were calculated from Kruskal–Wallis test followed by Dunn’s post hoc test. HS group comparing to JJ group: * *p* < 0.05, and **** *p* <0.0001; JS group comparing to JJ group: ^##^ *p* < 0.01, and ^####^ *p* <0.0001.

**Table 3:** Antioxidant capacities of the ethanolic extracts in *C. lanceolata*.

Assays	*C. lanceolata*			*p*-Value
	JJ	HS	JS	
DPPH radical scavenging assay (IC_50_ mg/mL)	20.04 ± 5.72	13.89 ± 2.03 ***	15.31 ± 2.59 ^#^	0.0006
FRAP assay (µmol FE/g dry weight)	22.53 ± 7.12	32.06 ± 6.55 ***	31.22 ± 6.05 ^###^	<0.0001
Antioxidant assay (µmol TE/g dry weight)	8.19 ± 3.02	9.40 ± 2.05	10.14 ± 2.60	0.092

The results are presented as mean ± SD. *p*-values were calculated from Kruskal–Wallis test followed by Dunn’s post hoc test. HS group comparing to JJ group: *** *p* <0.001; JS group comparing to JJ group: ^#^ *p* < 0.05, and ^###^ *p* <0.001.

## Data Availability

Data is contained within the article or supplementary material.

## Supplementary Materials

The following supporting information can be downloaded at: https://www.mdpi.com/article/10.3390/foods12020267/s1. The following are available online at Table S1. Metabolites identified by UPLC-QTOF-MS in *C. lanceolata* extracts; Figure S1. Geographical locations and representative samples of *C. lanceolata*; Figure S2. Total ion chromatograms of *C. lanceolata* extracts under positive ionization (A) and negative ionization (B); Figure S3. PCA score plot derived from the spectra of the positive (A) and negative (B) mode of UPLC-QTOF-MS in *C. lanceolata* extracts obtained from three different geographical origins.
