# Supplementary material for: UPLC-QTOF-MS-Based Metabolomics and Antioxidant Capacity of Codonopsis lanceolata from Different Geographical Origins
Source: Foods. 2023 Jan 6;12(2):267. doi: 10.3390/foods12020267 (PMC9858319; doi:10.3390/foods12020267)
Supplement: Supplementary file 1 [file foods-12-00267-s001.zip › foods-2084106-supplementary.pdf]

UPLC-QTOF-MS-based metabolomics and antioxidant capacity of *Codonopsis lanceolata* from different geographical origins

Miso Nam[a], Sae rom Jo[a], Young-Chan Kim[b], and Min-Sun Kim[a,]*

[a]Food Analysis Research Center, Korea Food Research Institute, Republic of Korea

[b]Enterprise Solution Research Center, Korea Food Research Institute, Republic of Korea

* Corresponding Authors

E-mail addresses: mskim@kfri.re.kr (M.S. Kim).

Table S1. Metabolites identified by UPLC-QTOF-MS in *C. lanceolata* extracts

No.	Metabolite	RT (min)	Adduct	Precursor ion (*m/z*)		difference (ppm)	Formula	Fragment ion	Fold change	
				Theoretical	observed				JS/JJ	HS/JJ
					**Organic acid**					
1	alpha-Ketoglutarate	0.61	[M-H]-	145.0142	145.0137	3.45	C5H6O5	127	0.85	0.87
2	Azelaic acid	4.98	[M-H]-	187.0976	187.097	3.21	C9H16O4	117, 125	0.70	0.78
3	Citrate	1.39	[M-H]-	191.0197	191.0181	8.38	C6H8O7	87, 111	0.99	1.05
4	Ascorbic acid	0.72	[M-H]-	175.0248	175.0237	6.28	C6H8O6	101, 115, 113	0.91	0.95
5	Fumarate	0.7	[M-H]-	115.0037	115.0025	10.43	C4H4O4	71	0.82	0.82
6	Malate	0.7	[M-H]-	133.0142	133.0128	10.53	C4H6O5	71, 73, 115	0.82	0.84
7	Succinate	0.76	[M-H]-	117.0193	117.0178	12.82	C4H6O4	73	0.68	0.67
8	Dihydrojasmonic acid	5.24	[M+H]+	213.1485	213.1474	5.16	C12H20O3	71, 107, 125, 171, 195	0.74	0.49
9	Aconitic acid	0.71	[M-H]-	173.0092	173.0090	1.16	C6H6O6	85, 111, 129, 155	0.95	1.07
10	Quinic acid	3.48	[M-H]-	191.0561	191.0563	1.05	C7H12O6	85, 93	0.98	2.01
11	Shikimic acid	3.53	[M-H]-	173.0455	173.0452	1.73	C7H10O5	129	0.92	3.32
					**Amino acid and derivatives**					
12	Arginine	0.54	[M+H]+	175.119	175.1176	7.99	C6H14N4O2	60, 70, 116, 130, 158	0.71	0.90
13	Aspartate	0.52	[M-H]-	132.0302	132.0297	3.79	C4H7NO4	71, 88, 115	0.64	0.75
14	Glutamate	0.55	[M+H]+	148.0604	148.0601	2.03	C5H9NO4	84, 102, 130	0.96	0.84
15	Glutamine	0.57	[M+H]+	147.0764	147.0766	1.36	C5H10N2O3	84	0.96	0.66
16	Histidine	0.5	[M-H]-	154.0622	154.0613	5.84	C6H9N3O2	67, 80, 93, 137	0.64	0.86
17	Leucine / Isoleucine	0.77	[M+H]+	132.1019	132.1011	6.06	C6H13NO2	57, 69, 86	0.58	0.70
18	Lysine	0.47	[M+H]+	147.1128	147.1124	2.72	C6H14N2O2	84	0.42	0.72
19	Methionine	0.72	[M+H]+	150.0583	150.0586	2.00	C5H11NO2S	56, 61, 104, 133	0.84	0.68
20	Ornithine	0.51	[M-H]-	131.0826	131.0817	6.87	C5H12N2O2	70, 72, 114	0.67	0.89
21	Phenylalanine	2.75	[M-H]-	164.0717	164.0709	4.88	C9H11NO2	103, 147	0.60	0.70

22	Proline	0.61	[M+H]+	116.0706	116.0701	4.31	C5H9NO2	70	0.65	0.89
23	Serine	0.57	[M+H]+	106.0499	106.0498	0.94	C3H7NO3	74	0.59	0.58
24	Threonine	0.57	[M+H]+	120.0655	120.0658	2.50	C4H9NO3	102, 120	0.89	0.70
25	Tryptophan	3.24	[M-H]-	203.0826	203.0809	8.37	C11H12N2O2	116, 159	0.67	0.78
26	Tyrosine	2.12	[M+H]+	182.0812	182.0807	2.75	C9H11NO3	136	0.87	1.00
27	Valine	0.7	[M+H]+	118.0863	118.0861	1.69	C5H11NO2	72	0.76	0.69
28	Dimethylarginine	0.66	[M+H]+	203.1503	203.1497	2.95	C8H18N4O2	116, 158	0.76	0.95
29	Choline	0.56	[M]+	104.1075	104.1061	13.45	C5H14NO	58, 59, 60	1.04	0.95
30	Acetylcholine	0.65	[M]+	146.1181	146.1169	8.21	C7H16NO2	58, 87	2.05	1.03
31	Phosphocholine	0.55	[M]+	184.0739	184.0746	3.80	C5H15NO4P	88	1.22	1.04
32	Pyroglutamate	0.57	[M+H]+	130.0499	130.0501	1.54	C5H7NO3	56, 84	0.91	0.68
33	Betaine	0.57	[M+H]+	118.0863	118.0860	2.54	C5H11NO2	58, 59	1.39	1.02
34	Glutathione	1.33	[M+H]+	308.0911	308.0905	1.95	C10H17N3O6S	179, 233	0.37	0.75
35	Carnitine	0.59	[M+H]+	162.1125	162.1125	0.00	C7H15NO3	57, 58, 60, 85, 102, 103	2.20	1.16
					**Sugar**					
36	Raffinose	0.68	[M-H]-	503.1618	503.1595	4.57	C18H32O16	59, 89, 119,161, 179, 323, 341	0.59	0.75
37	Sucrose	0.67	[M-H]-	341.1089	341.1084	1.47	C12H22O11	161, 179	0.75	0.79
38	Glucose	0.57	[M-H]-	179.0561	179.0554	3.91	C6H12O6	161	0.54	0.67
					**Glycoside**					
39	Geniposide	3.35	[M-H]-	387.1297	387.1285	3.10	C17H24O10	146, 161, 164, 179	0.68	1.34
40	Woodorien	2.92	[M-H]-	329.0878	329.0875	0.91	C14H18O9	147, 164, 167	1.18	1.04
					**Triterpenoids**					
41	Echinocystic acid	7.12	[M+H]+	473.3625	473.3625	0.00	C30H48O4	427, 437, 455	1.16	0.94
42	Lancemaside A	7.14	[M-H]-	1189.5648	1189.5628	1.68	C57H90O26	647	0.85	0.85
43	Lancemaside B	6.38	[M-H]-	1351.6176	1351.6131	3.33	C63H100O31	647	0.97	0.80
44	Lancemaside C	6.92	[M-H]-	1219.5753	1219.5727	2.13	C58H92O27	647	1.36	0.90

45	Lancemaside D	6.64	[M-H]-	1087.5331	1087.5293	3.49	C53H84O23	647	1.62	1.05
46	Lancemaside E	6.74	[M-H]-	1351.6176	1351.6148	2.07	C63H100O31	809	1.92	1.15
47	Lancemaside G	6.42	[M-H]-	1205.5596	1205.5569	2.24	C57H90O27	663	0.91	0.93
48	Aster saponin Hb	7.24	[M-H]-	925.4802	925.4777	2.70	C47H74O18	647	4.60	2.24
49	Foetidissimoside A	7.08	[M-H]-	1057.5225	1057.5178	4.44	C52H82O22	647	3.00	2.44
50	Codonolaside I	7.59	[M-H]-	1203.5804	1203.5761	3.57	C58H92O26	647	1.44	0.95
51	Codonolaside II	9.49	[M-H]-	1013.5326	1013.5353	2.66	C51H82O20	469, 541	1.32	0.86
					**Polyacetylene**					
52	Lobetyol	4.69	[M-H]-	233.1170	233.1170	5.58	C14H18O3	103, 133, 145	0.67	0.99
53	Lobetyolin	5.02	[M+HCOO]-	441.1757	441.1757	2.04	C20H28O8	143, 159, 185, 215	0.84	0.89
54	Lobetyolinin	4.53	[M+HCOO]-	603.2280	603.2280	2.32	C26H38O13	179, 323, 221, 341, 467	0.84	0.98
55	Lobetyolinin sulfate	0.69	[M-H]-	637.1822	637.1822	2.35	C26H38O16S	71, 115, 133	0.69	0.82
56	Capillene	5.02	[M+H]+	155.0856	155.0856	0.64	C12H10	115, 127, 153	0.93	0.94
					**Phenylpropanoid**					
57	Syringin	3.56	[M+NH4]+	390.1741	390.1741	4.61	C17H24O9	105, 133, 161, 193	1.59	2.14
58	Tangshenoside I	3.91	[M-H]-	677.2278	677.2278	2.95	C29H42O18	261, 453, 497	1.05	1.22
59	Tangshenoside II	3.56	[M+HCOO]-	417.1390	417.1390	2.88	C17H24O9	194, 209	1.33	1.60
60	Tangshenoside IV	4.34	[M-H]-	1031.3563	1031.3563	4.85	C46H64O26	261, 383, 545, 645, 807, 851	3.80	5.37
61	Tangshenoside VIII	4.53	[M+Na]+	1055.3590	1055.359	0.66	C46H64O26	193, 553, 701	2.64	5.22
62	6-Methylcoumarin	3.9	[M+H]+	161.0587	161.0587	6.21	C10H8O2	105, 115, 118, 133	1.49	1.91
63	Myristicin	3.9	[M+H]+	193.0845	193.0845	7.25	C11H12O3	105, 115, 133, 161	1.38	1.65
64	Salicylic acid	3.15	[M-H]-	137.0233	137.0233	8.03	C7H6O3	65, 93, 95, 121	0.86	0.75
65	Scopoletin	3.16	[M+H]+	193.0483	193.0483	6.22	C10H8O4	133	0.89	1.06
66	Phenylacetate	3.14	[M-H]-	135.0445	135.0445	5.18	C8H8O2	93	0.84	0.85
67	Ferulic acid	3.36	[M-H]-	193.0495	193.0495	5.70	C10H10O4	134	0.98	2.79
68	Gallic acid	2.67	[M+H]+	171.0284	171.0284	2.34	C7H6O5	153	1.72	0.69

69	Coumaric acid	4.1	[M+H]+	165.0542	165.0542	2.42	C9H8O3	91, 119, 147	0.69	1.06
70	Gentisic acid	2.87	[M+H]+	155.0331	155.0331	5.16	C7H6O4	81, 137	1.91	0.77
71	7-Hydroxycoumarin	3.47	[M+H]+	163.0388	163.0388	1.23	C9H6O3	77, 89, 145	1.02	2.25
72	Caffeic acid	3.15	[M-H]-	179.0348	179.0348	1.12	C9H8O4	59, 93, 135	0.80	0.80
73	3-Hydroxybenzoic acid	2.56	[M+H]+	139.0382	139.0382	5.75	C7H6O3	77, 95, 121	1.13	0.96
74	Protocatechuic acid	3.26	[M-H]-	153.0185	153.0185	5.23	C7H6O4	109, 135	1.07	0.69
75	Chlorogenic acid	3.48	[M-H]-	353.0870	353.0870	2.27	C16H18O9	191	0.99	2.19
76	Cinnamaldehyde	3.91	[M+H]+	133.0638	133.0638	7.52	C9H8O	77, 79, 103, 105, 115	1.50	1.97
77	Cinnamic acid	2.75	[M-H]-	147.0442	147.0442	6.80	C9H8O2	103, 131	0.62	0.71
					**Flavonoid**					
78	Kaempferol-O-glucoside	4.38	[M+H]+	449.1078	449.1064	3.12	C21H20O11	287	2.24	2.27
					**Others**					
79	Tianshi acid	8.16	[M-H]-	329.2333	329.2339	1.82	C18H34O5	171, 211, 229	0.97	0.86
80	4-Methylnicotinic acid	0.63	[M+H]+	138.0550	138.0546	2.90	C7H7NO2	65, 78, 92, 120	0.80	0.88
81	Adenosine	2.43	[M+H]+	268.1040	268.1040	0.00	C10H13N5O4	136	0.88	0.89
82	Pantothenic acid	2.93	[M+H]+	220.1179	220.1170	4.09	C9H17NO5	90, 98, 116, 124, 184, 202	0.71	0.74
83	Indole	3.25	[M+H]+	118.0651	118.0642	7.62	C8H7N		0.77	0.81
84	2-Methylindole	3.25	[M+H]+	132.0808	132.0811	2.27	C9H9N	105, 117	0.77	0.81
85	(E)-2-Hexenyl-α-L-arabinopyranosyl-(1→2)-β-D-glucopyranoside	4.16	[M-H]-	393.1766	393.1760	1.53	C17H30O10	261, 149	3.69	2.81
86	6-Methylquinoline	3.26	[M+H]+	144.0808	144.0800	5.55	C10H9N	91, 117	0.77	0.81
87	Benzaldehyde	2.77	[M+H]+	107.0491	107.0488	2.80	C7H6O	77, 79	0.68	0.75
88	Hexyl-β-D-glucopyranosyl-(1→2)-β-D-glucopyranoside	4.23	[M-H]-	425.2028	425.2023	1.18	C18H34O11	101, 263	2.81	1.18
89	Hexyl-β-D-glucoside sulfate	3.09	[M-H]-	343.1068	343.1028	11.66	C12H24O9S	163	1.35	0.88
90	Indoleacrylic acid	3.25	[M+H]+	188.0706	188.0697	4.79	C11H9NO2	170	0.79	0.82
91	Indolelactic acid	3.62	[M+H]+	206.0812	206.0805	6.40	C11H11NO3	118, 130, 160, 188	1.08	1.02
92	Codonopyrrolidium A	4.46	[M+HCOO]-	395.1949	395.1895	13.66	C19H28NO5	101, 131, 161, 263	3.02	1.84

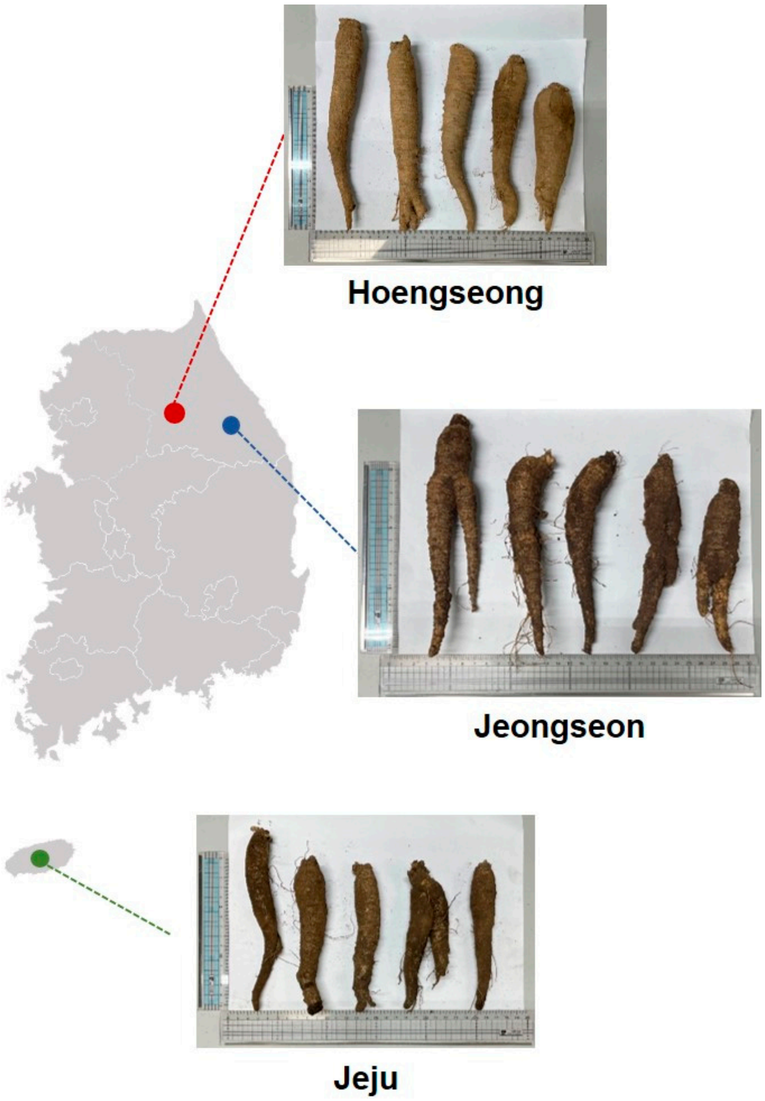

Figure S1. Geographical locations and representative samples of C. lanceolata.

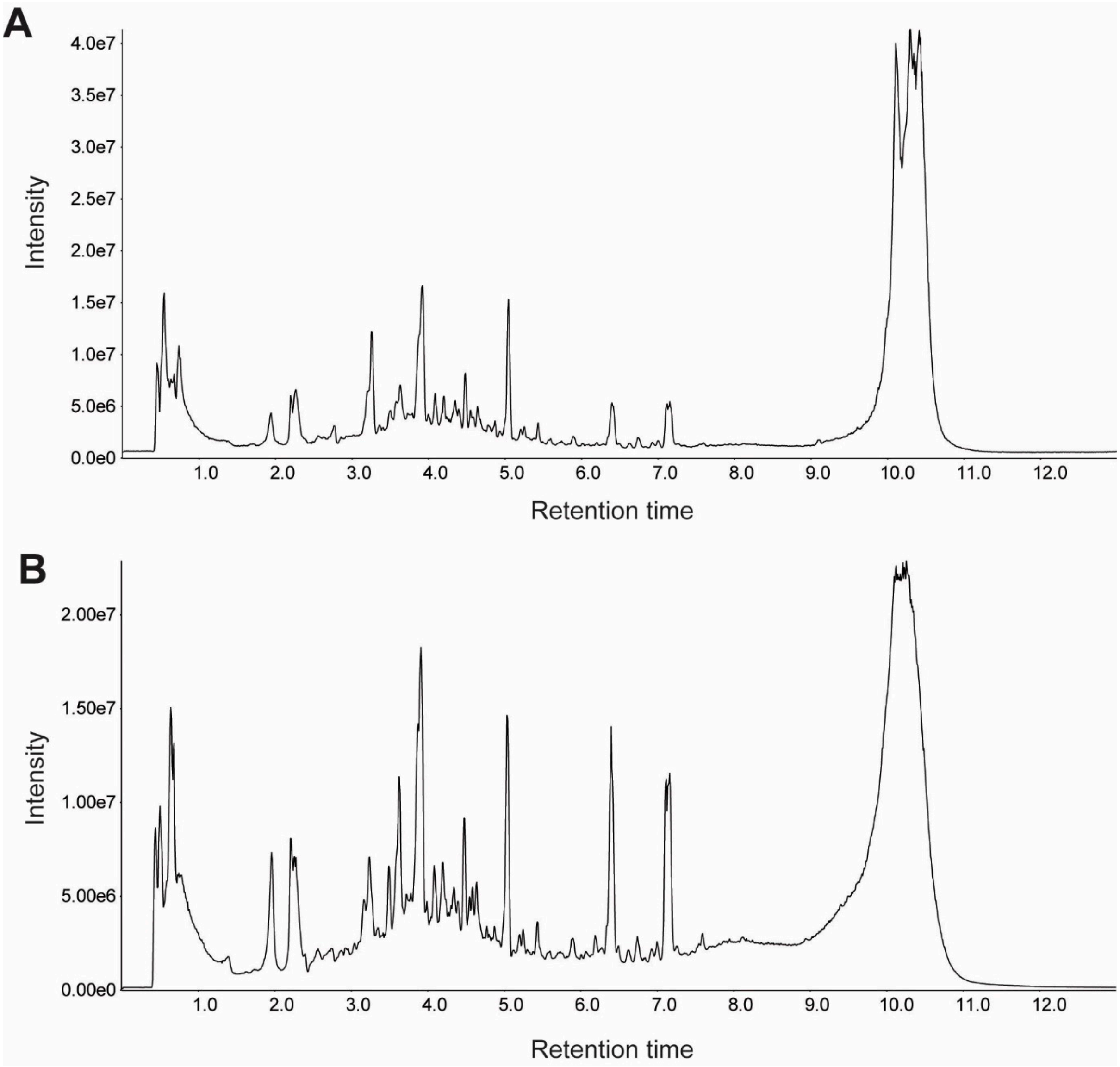

Figure S2. Total ion chromatograms of *C. lanceolata* extracts under positive ionization (A) and negative ionization (B).

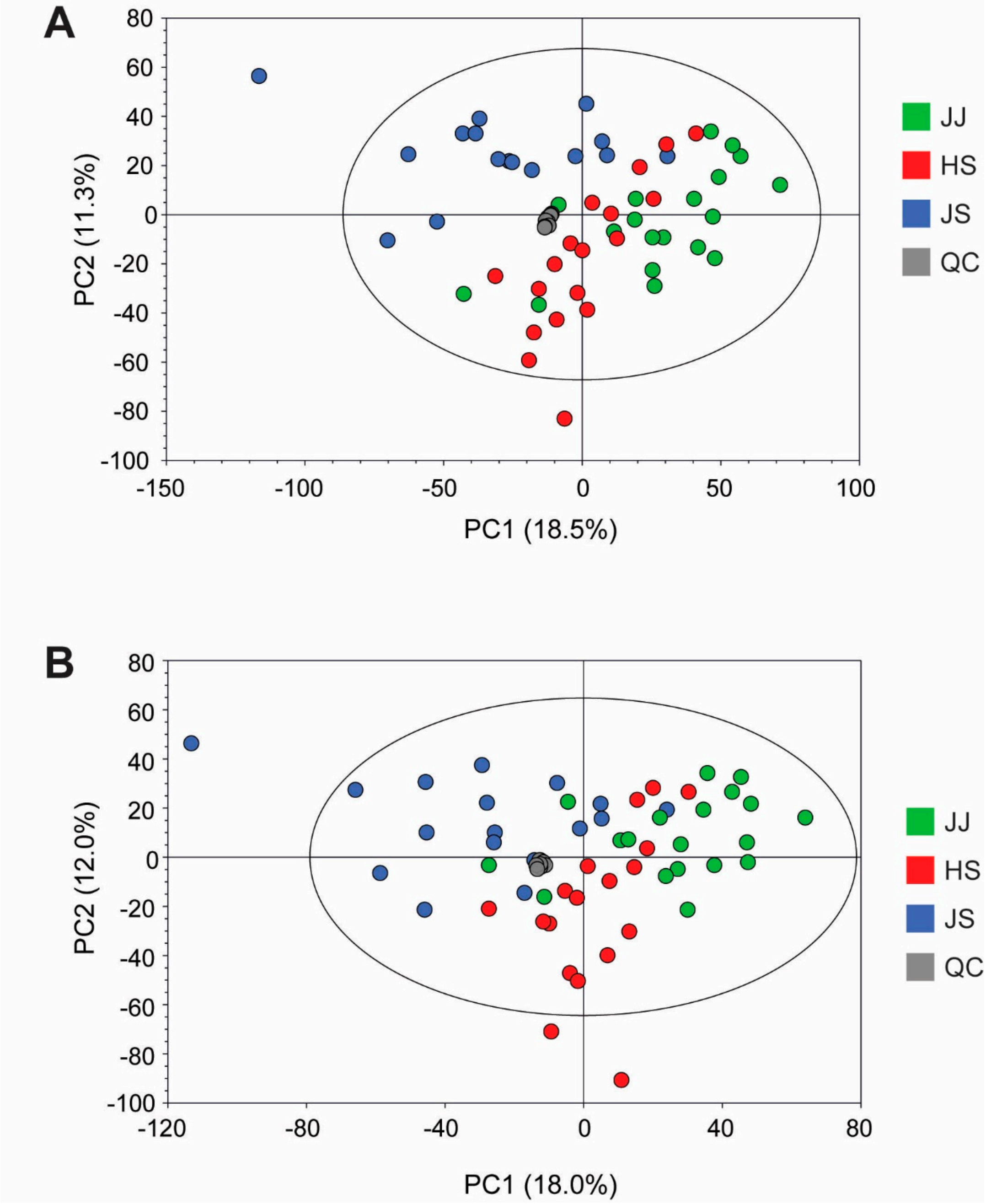

Figure S3. PCA score plot derived from the spectra of the positive (A) and negative (B) mode of UPLC-QTOF-MS in *C. lanceolata* extracts obtained from three different geographical origins.
